# Pathological response to pembrolizumab-based neoadjuvant therapy in ER-low vs. ER-zero breast cancer: a Swedish population-based cohort study

**DOI:** 10.1186/s13058-025-02179-3

**Published:** 2025-11-29

**Authors:** Sanna Steen, Emelie Karlsson, Ida Björnheden, Gunilla Rask, Viktoria Thurfjell, Hampus Nobin, Blanka Kolodziej, Anna Bodén, Annette Bauer, Rickard Einefors, Per Nilsson, Ioannis Zerdes, Andri Papakonstantinou, Theodoros Foukakis, Irma Fredriksson, Mattias Rantalainen, Eugenia Colón-Cervantes, Anikó Kovács, Balazs Acs, Johan Hartman

**Affiliations:** 1 Department of Oncology-Pathology, Karolinska Institutet, Stockholm, Sweden; 2 Department of Clinical Pathology and Cancer Diagnostics, Karolinska University Hospital, Stockholm, Sweden; 3 Medical Unit Nuclear Medicine and Hospital Physics, Karolinska University Hospital, Stockholm, Sweden; 4 Unit of Clinical Genetics, Pathology and Molecular Diagnostics, Skåne University Hospital, Malmö, Sweden; 5 Department of Pathology, Umeå University Hospital, Umeå, Sweden; 6 Department of Diagnostics and Intervention, Surgery, Umeå University, Umeå, Sweden; 7 Department of Clinical Pathology, Uppsala University Hospital, Uppsala, Sweden; 8 Department of Immunology, Genetics and Pathology, Uppsala University, Uppsala, Sweden; 9 Department of Pathology, Kalmar County Hospital, Region Kalmar, Kalmar, Sweden; 10 Department of Clinical Sciences Lund, Division of Pathology, Lund University, Lund, Sweden; 11 Department of Laboratory Medicine and Pathology, Region Jönköping County Hospital, Jönköping, Sweden; 12 Department of Clinical Pathology, Department of Biomedical and Clinical Sciences, Linköping University, Linköping, Sweden; 13 Centre for Medical Image Science and Visualization, Linköping University, Linköping, Sweden; 14 Department of Pathology and Cytology Dalarna, County Hospital Dalarna, Falun, Sweden; 15 Department of Laboratory Medicine, Örebro University Hospital, Örebro, Sweden; 16 Department of Pathology, Växjö Central Hospital, Region Kronoberg, Sweden; 17 Breast Center, Theme Cancer, Karolinska University Hospital, Stockholm, Sweden; 18 Department of Molecular Medicine and Surgery, Karolinska Institutet, Stockholm, Sweden; 19 Department of Breast, Endocrine Tumors and Sarcoma, Karolinska University Hospital, Stockholm, Sweden; 20 Department of Medical Epidemiology and Biostatistics, Karolinska Institutet, Stockholm, Sweden; 21 Department of Medical Epidemiology and Biostatistics, Karolinska Institutet, Stockholm, Sweden; 22 Department of Clinical and Surgical Pathology, Unilabs, Stockholm, Sweden; 23 Department of Clinical Pathology, Sahlgrenska University Hospital, Gothenburg, Sweden; 24 Institute of Biomedicine, Sahlgrenska Academy, University of Gothenburg, Gothenburg, Sweden; 25 MedTechLabs, Karolinska University Hospital, BioClinicum NKS J5:20, Solnavägen 30, 171 64 Bioclinicum, Solna, Sweden

**Keywords:** Triple negative breast cancer, Neoadjuvant immunotherapy, Pembrolizumab, ER low

## Abstract

**Background:**

Emerging evidence indicates that estrogen receptor-low (ER-low)/human epidermal growth factor receptor 2 negative (HER2-) breast cancer (BC) may more closely resemble ER-negative (ER-zero, < 1%) rather than ER-positive disease in terms of biological and clinicopathological characteristics. In Sweden, ER-low (ER 1–9%) BC is managed as triple-negative breast cancer (TNBC) and is thus eligible for neoadjuvant chemo-immunotherapy. We aimed to investigate real-world pathological response to neoadjuvant pembrolizumab combined with chemotherapy in ER-low versus ER-zero BC patients within a Swedish population-based multi-center cohort.

**Methods:**

BC patients with indication to receive neoadjuvant pembrolizumab in combination with chemotherapy in Sweden between 2022 and 2024 were included in the study. Clinicopathological data—including pathological complete response (pCR) status, residual cancer burden (RCB) score, stromal tumor-infiltrating lymphocytes (sTILs) levels, and routine tumor characteristics—were retrieved from laboratory information systems. Associations between categorical variables were assessed using chi-squared (χ^2^) tests and associations between continuous variables and ER status or pCR were analyzed using Mann–Whitney U-test.

**Results:**

The total cohort comprised 441 TNBC cases (ER-zero n = 398; ER-low n = 43). In the ER-zero group, the pCR rate and RCB score 0–1 were 50.5% (95% CI: 45.5% to 55.5%) and 60.8% (95% CI: 55.8% to 65.6%), respectively. In the ER-low group, the corresponding values were 58.1% (95% CI: 42.1% to 73%), and 60.5% (95% CI: 44.4% to 75%), respectively. There were no statistically significant differences in either pCR rate (*p* = 0.46) or dichotomized RCB score (*p* = 0.88) between the groups. The ER-low group showed significantly higher sTILs percentage compared to the ER-zero group (median sTILs 25% versus 20%, *p* = 0.046). However, when sTILs were analyzed as a binary categorical variable using a 30% cut-off, no significant difference was observed (*p* = 0.33).

**Conclusions:**

We observed no significant difference in pathological response to neoadjuvant chemo-immunotherapy with pembrolizumab between ER-zero and ER-low BCs. These findings support previous evidence suggesting that ER-low tumors behave more similarly to ER-zero than ER-positive.

**Supplementary Information:**

The online version contains supplementary material available at 10.1186/s13058-025-02179-3.

## Background

Triple-negative breast cancer (TNBC) accounts for 10–15% of all breast cancers (BC) and is defined by the absence of estrogen receptor (ER), progesterone receptor (PR) and human epidermal growth factor receptor 2 (HER2) expression. The immunohistochemistry-based classification of BC into surrogate intrinsic subtypes was established based on hierarchical clustering of gene expression profiles generated from cDNA microarray analyses and subsequently refined through the development of the Prediction Analysis of Microarray (PAM50) classifier [1–3]. TNBC is a biologically aggressive subtype, associated with high proliferation and early relapse, resulting in poor prognosis compared to other BC surrogate intrinsic subtypes [4]. TNBC is the most immunologically active form of BCs, characterized by abundant tumor-infiltrating lymphocytes (TILs), indicating a potentially favorable response to immunotherapy. For high-risk, early-stage TNBC, chemotherapy in combination with neoadjuvant pembrolizumab, an immune checkpoint inhibitor, has been shown to significantly improve pathological complete response (pCR) rates and long-term outcomes [5]. Despite its clinical definition, TNBC is biologically heterogenous and can be further stratified by gene expression profiling into several molecular subtypes with different ontologies and response to treatment [6–8].

ER-low BCs, as defined in 2020 by American Society of Clinical Oncology (ASCO) guideline update,^9^ represent a therapeutic challenge due to their biological heterogeneity and inconsistent treatment response [10]. Emerging evidence indicates that ER-low tumors may more closely resemble ER-negative (ER-zero, < 1%) tumors, rather than ER-positive disease in terms of biological and clinicopathological characteristics [11–16]. Gene expression profiling has shown very few luminal cases in the group of ER-low [17]. Swedish BC patients with ER-low/HER2-negative (ER-low/HER2-) disease have generally been treated as TNBC, resulting in a broader and more heterogeneous patient population compared to international cohorts. This approach is based on the Swedish Breast Cancer Group’s decision to maintain the ≥ 10% cut-off for both ER and PR positivity, due to limited scientific evidence to support the ASCO and College of American Pathologists (CAP) guidelines lowering the threshold to ≥ 1% [18]. A deeper understanding of the clinical relevance of ER-low versus ER-zero status is essential to refining patient selection and to optimize therapeutic strategies.

According to Swedish guidelines, TNBC is defined by the lack of ER and PR expression < 10%, and HER2 negativity, assessed as either IHC 0–1+, or IHC 2 + without amplification by in situ hybridization (ISH) [19]. Since 2022, the Swedish Breast Cancer Treatment Guidelines have endorsed the KEYNOTE-522 (KN522) regimen, consisting of neoadjuvant pembrolizumab in combination with chemotherapy (chemo-immunotherapy), followed by adjuvant pembrolizumab for patients with TNBC and locally advanced disease (T3-T4) or tumor size > 2 cm, and/or confirmed axillary lymph node metastases [19]. According to the same guidelines, Ki67 index scoring is mandatory for all breast cancers to improve risk stratification, while TILs assessment is strongly recommended for all TNBCs, although the clinical utility remains uncertain [19]. 

This study aimed to investigate real-world pathological response to neoadjuvant pembrolizumab in addition to chemotherapy in ER-low versus ER-zero breast cancer in a Swedish population-based cohort.

## Methods

### Swedish neoadjuvant pembrolizumab plus chemotherapy breast cancer cohort 2022–2024

The study was designed as a multi-center retrospective cohort study, aiming to reflect a population-representative real-world clinical setting. All Swedish BC patients with indication to receive neoadjuvant pembrolizumab in combination with chemotherapy during the period from January 1, 2022, to December 31, 2024, reported to the Swedish National Quality Register for Breast Cancer were included in the study. The Swedish breast cancer register is a nationwide registry encompassing approximately 99% of all BC patients in Sweden, providing high-quality data with virtually complete follow-up [20].dSweden has a tax-funded healthcare system, including a national breast cancer screening program for women aged 40–75. Data was extracted from the register on February 25th, 2025. Due to delays in reporting treatment data, the number of patients treated in late 2024 cannot be considered complete at the time of data extraction.

Designated pathologists at each participating hospital were responsible for retrieving clinicopathological data from their respective Laboratory Information Systems (LIS). These data comprised pCR defined as the absence of invasive carcinoma in both breast and axillary lymph node tissue after neoadjuvant chemo-immunotherapy; Residual Cancer Burden (RCB) score; size of the largest invasive tumor focus in the resected specimen; lymph node involvement in surgical specimen; stromal tumor-infiltrating lymphocytes (sTILs) assessed in the treatment-naïve biopsy; tumor grade; histological type; and immunohistochemical (IHC) biomarker percentage for ER, PR, Ki67 and HER2, as well as HER2 ISH results when applicable. After data collection, cases with identified HER2-positive breast cancer, ER-positive breast cancer (≥ 10%), or cases missing pathology report numbers were excluded (Table 1).

### Statistical methods

Clinicopathological characteristics were summarized using descriptive frequencies and presented as percentages. In the summary table (Table 1), the continuous variables ER and PR were categorized into clinically relevant groups: negative (< 1%), low (1–9%), and positive (≥ 10%). Ki67 percentages were categorized according to cut-off values applied in Swedish clinical practice, [19] based on recommendations from the International Ki67 in Breast Cancer Working Group (IKWG) [21]: high (> 29%), intermediate (6–29%), and low (< 6%). All HER2 cases had IHC scores of either 0–1 + or IHC 2 + with non-amplified ISH results and were therefore categorized as negative. For sTILs, a cut-off value of 30% was applied, based on the study by Park et al., which demonstrated excellent survival outcomes in early TNBC with sTILs ≥ 30% [22]. Pathological characteristics were stratified by ER status (ER-zero versus ER-low), and group differences were analyzed using chi-squared (c^2^) test and Fisher’s exact test, as all variables were categorical or categorized. Associations between categorical variables and treatment response were analyzed using chi-squared (c^2^) test. Associations between continuous variables and ER status as well as pCR were analyzed using Mann-Whitney U-test. A multivariable logistic regression model was applied to investigate the adjusted association between ER-low/ER-zero status and pCR (further details are provided in Supplementary Materials Table S4).

All analyses were conducted using R version 4.4.3. All statistical tests were two-sided, with a significance level of 5% (*p*-value < 0.05).

## Results

**Fig. 1:**
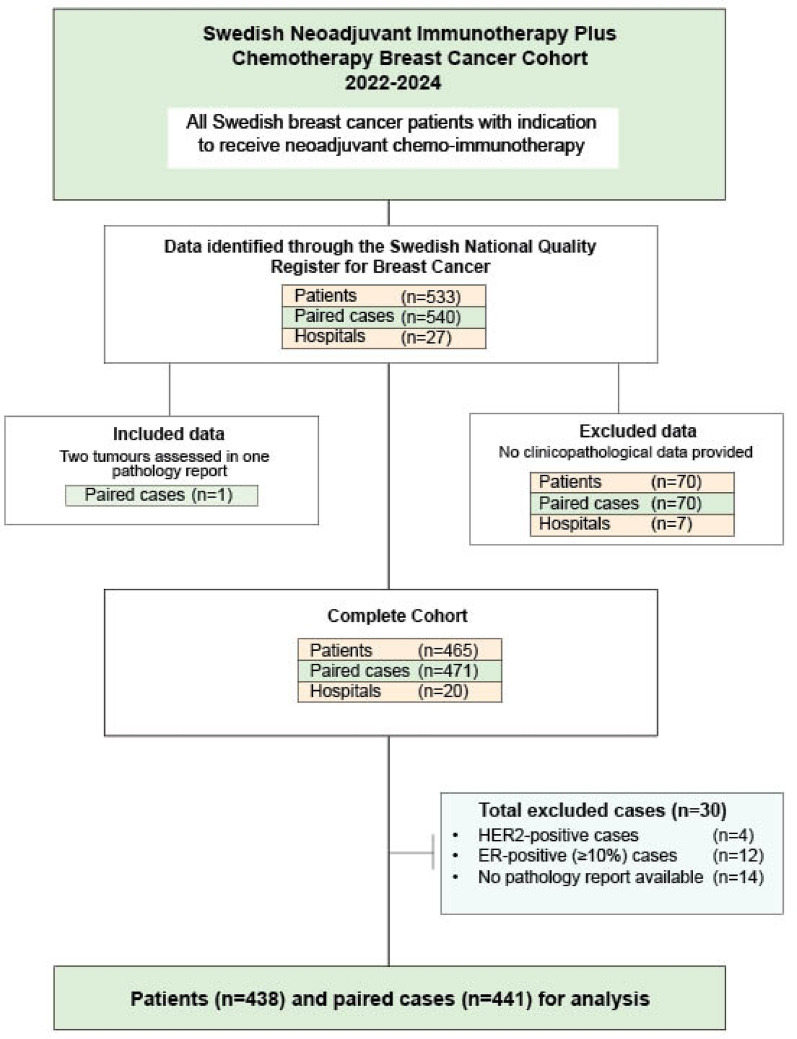
Consort diagram of the patient selection of the Swedish neoadjuvant pembrolizumab plus chemotherapy breast cancer cohort 2022–2024. HER2 = Human epidermal growth factor receptor 2; ER = estrogen receptor

Our patient cohort with inclusion and exclusion criteria is summarized in Fig. 1. Through the Swedish breast cancer register, 540 paired cases (diagnostic biopsy and corresponding surgical specimen) from 533 BC patients intended to receive neoadjuvant pembrolizumab and chemotherapy at 27 Swedish hospitals were identified. Clinicopathological data were obtained from 20 hospitals, including a mix of hospitals from regional university hospitals (e.g., in Stockholm, Gothenburg and Malmö), county hospitals (e.g., Jönköping and Falun) and private hospitals (e.g., Capio S: t Göran’s hospital in Stockholm), comprising 471 paired cases from 465 patients. One additional case was included owing to two tumors being included in one pathology report. Of the 471 paired cases, the following were excluded: HER2-positive breast cancer (*n* = 4), ER-positive breast cancer (≥ 10%) (*n* = 12), cases missing pathology report numbers in LIS (*n* = 13) and one patient deceased before surgery (*n* = 1). The final cohort comprised 441 paired TNBC from 438 BC patients, eligible for statistical analysis.

**Fig. 2:**
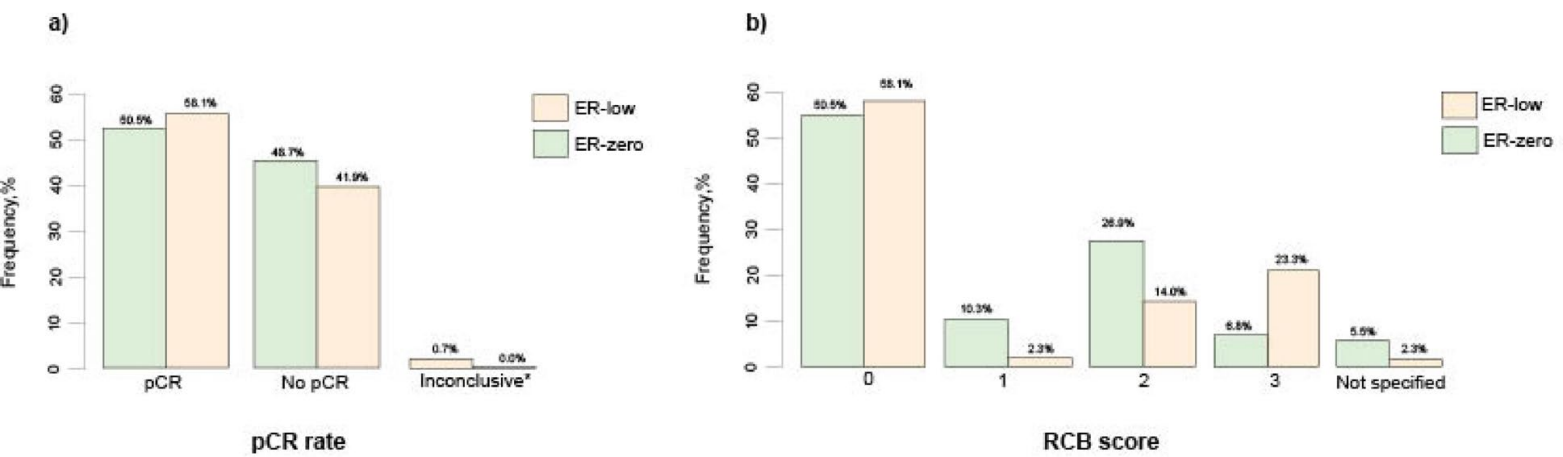
Bar charts illustrating the proportion of pathological complete response (pCR) rates (a) and residual cancer burden (RCB) scores (b) stratified by estrogen receptor (ER) status (ER-zero vs. ER-low). ER = estrogen receptor; pCR = pathological complete response; RCB = residual cancer burden. *The pathology report indicated the presence of a few cells that were difficult to assess

Descriptive statistics on clinicopathological data for the complete cohort of 441 paired cases are presented in Table 1. A total of 241 cases (54.6%) had no residual invasive tumor in the surgical specimen (Supplementary material Table S1), but 15 of those had residual nodal metastases, resulting in an overall pCR rate of 51.2%. sTILs count was reported in 76.4% of cases. Of the cases assessed, 398 were classified as ER-zero tumors and 43 as ER-low. When comparing ER-zero and ER-low patients, the pCR rate was 50.5% (95% CI: 45.5% to 55.5%) in the ER-zero group and 58.1% (95% CI: 42.1% to 73%), in the ER-low group, while the proportion of RCB score 0–1 was 60.8% (95% CI: 55.8% to 65.6%), and 60.5% (95% CI: 44.4% to 75%), respectively. ER-low patients demonstrated a slightly higher rate of PR positivity compared to ER-zero patients (*p* = 0.0013) (Table 1). ER status (ER-zero versus ER-low) was not significantly associated with either pCR or dichotomized RCB scores (*p* = 0.46 and *p* = 0.88, respectively) (Table 1; Fig. 2, and Supplementary material Table S2).

**Table 1:**
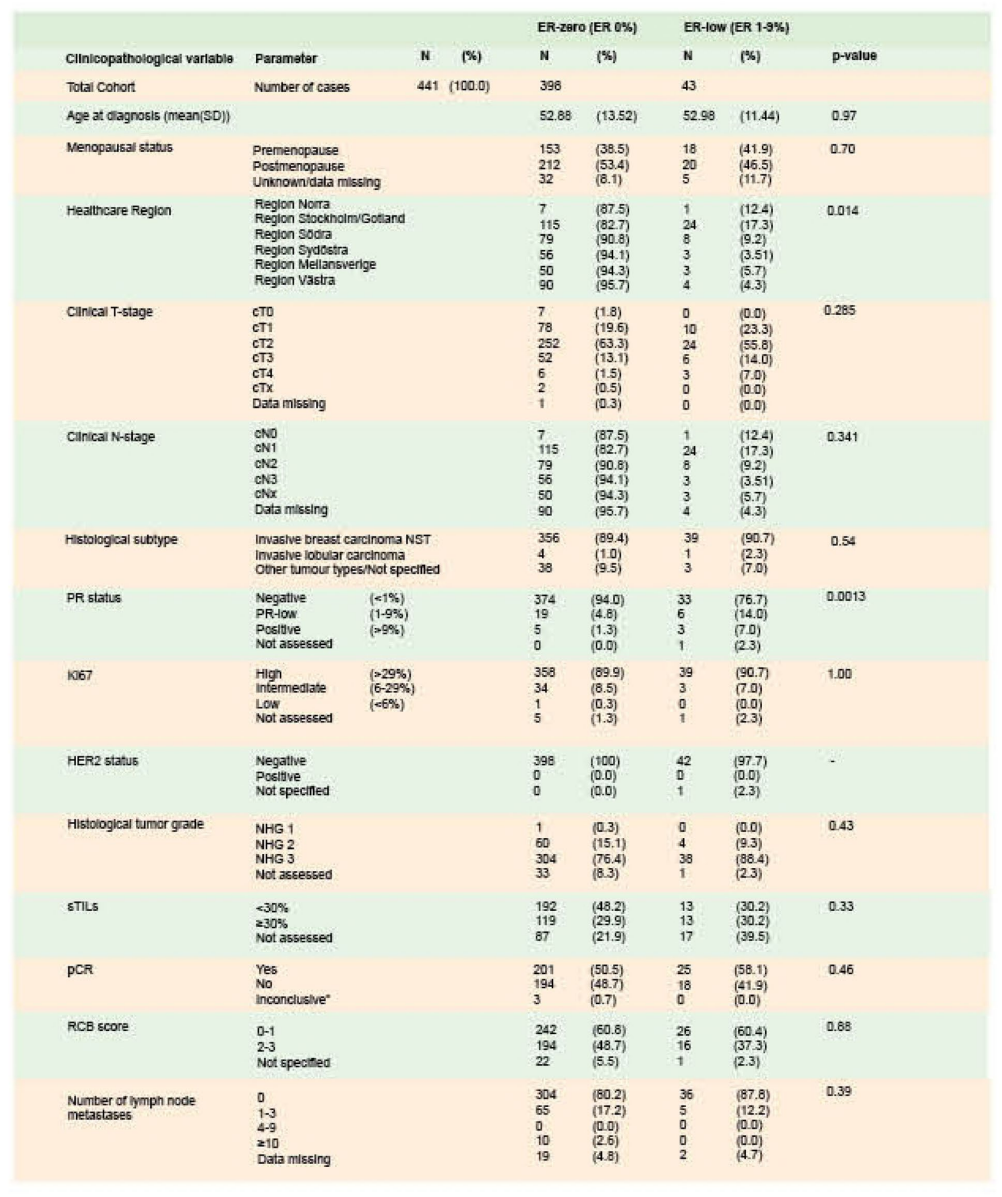
Clinicopathological characteristics stratified by Estrogen receptor (ER) status (ER-zero and ER-low)

ER = estrogen receptor; NST = no specific type; PR = progesterone receptor; Ki67 = Proliferation marker; HER2 = human epidermal growth factor receptor 2; NHG = Nottingham Histological Grade; sTILs = stromal tumor infiltrating lymphocytes; pCR = pathological complete response; RCB = residual cancer burden. * The pathology report indicated the presence of a few cells that were difficult to assess

**Fig. 3:**
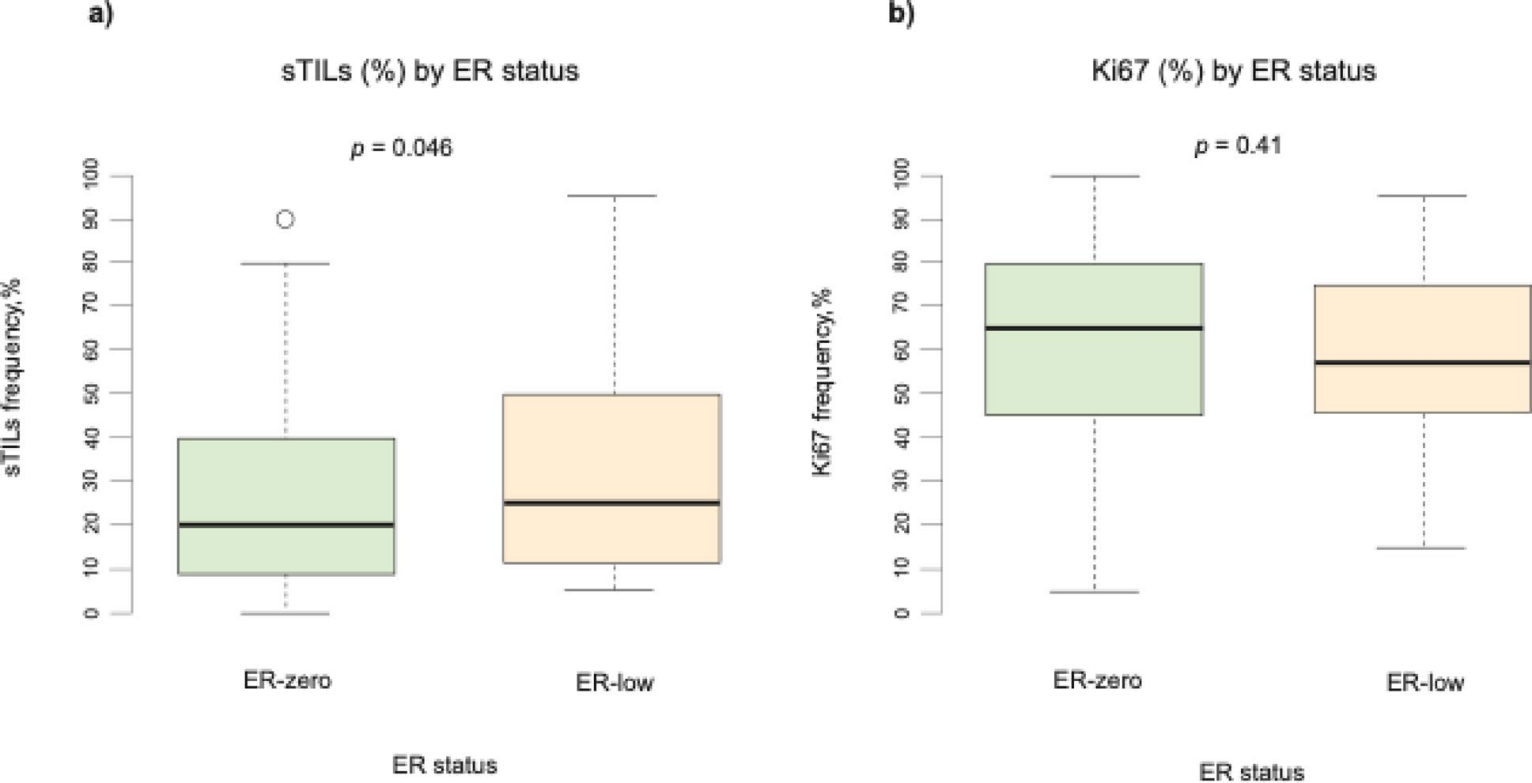
Boxplots illustrating continuous values of stromal tumor-infiltrating lymphocytes (sTILs) score (a) and proliferation marker (Ki67) index (b) stratified by estrogen receptor (ER) status (ER-zero versus ER-low groups) of the cohort. sTILs = stromal tumor-infiltrating lymphocytes; ER = estrogen receptor

A significant higher sTILs score was found in the ER-low group compared to ER-zero (median sTILs 25% versus 20%, respectively *p* = 0.046) (Fig. 3). However, when sTILs were analyzed as a binary categorical variable using a 30% cut-off, no significant difference was observed between ER-zero and ER-low (*p* = 0.33) (Table 2). No significant difference was observed in the continuous score for Ki67 comparing the two groups (Fig. 3). In subgroup analyses by pCR and non-pCR status, no significant differences were found between ER-zero and ER-low patient groups for either sTILs or Ki67 (Supplementary material Figure S1). As the distribution of histological subtype and histologic grade in the cohort was low, these variables were excluded from further analysis. Additionally, multivariable logistic regression analysis confirmed that ER-zero and ER-low status was not associated with pCR when adjusted for clinicopathological factors *(p* = 0.16) (Supplementary material Table S4 and Figure S2).

**Table 2:**
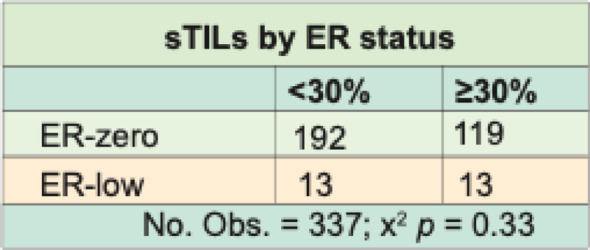
Crosstabulations of sTILs by ER status with chi-squared test results

sTILs = Stromal tumor-infiltrating lymphocytes; ER = estrogen receptor; No. = number; Obs. = observations

## Discussion

Neoadjuvant pembrolizumab has contributed substantially to the high pathological complete response rates observed in early TNBC demonstrated in the KN522 trial [23]. The KN522 study has changed clinical practice, and neoadjuvant pembrolizumab combined with chemotherapy has become the standard of care for patients with early-stage TNBC. Despite this significant benefit, the role of neoadjuvant pembrolizumab in ER-low/HER2-negative disease remains uncertain, as no contemporary randomized prospective trials have specifically investigated its efficacy in this subgroup. Following the 2010 revision of international guidelines redefining the cut-offs for estrogen and progesterone receptor positivity in breast cancer,^18^ the Swedish Breast Cancer Group (SweBCG) retained the ≥ 10% threshold. Consequently, the Swedish TNBC patient population has encompassed ER-low/HER2-negative breast cancer patients, which have generally also been treated as TNBC [24]. In light of the urgent clinical question regarding the efficacy of neoadjuvant pembrolizumab plus chemotherapy in ER-low BCs, and the unique treatment approach used in Sweden, we aimed to investigate real-world pathological response and clinicopathological tumor characteristics in ER-low versus ER-zero breast cancer patients, based on a nationwide Swedish population-based cohort, covering 99% of all Swedish BC patients. In our multi-center, population-based study, ER-low tumors exhibited pCR rates, and RCB and sTILs scores comparable to those observed in ER-zero TNBC patients. Furthermore, the pCR rates observed in our dataset are consistent with those reported in current literature of TNBC patients treated with neoadjuvant immuno-chemotherapy [25, 26]. 

Several recent studies have explored clinicopathological factors and molecular profiling in TNBC, including ER-low cases with neoadjuvant therapy [17, 27–31]. Findings suggest that the immune and biological landscape, including molecular intrinsic subtypes, of ER-low tumors more closely resembles that of ER-zero than ER-positive BCs [17, 28]. Recent studies have also reported pCR rates following neoadjuvant immuno-chemotherapy in ER-low patients that are consistent with our findings [27, 30]. Moreover, in line with our results, a recent study demonstrated that both sTILs and Ki67 are associated with pCR in early TNBC patients treated with neoadjuvant immuno-chemotherapy. However, these studies were either based on single-center patient cohorts, did not directly compare ER-low and ER-zero cases, or lacked correlation with neoadjuvant pembrolizumab plus chemotherapy [28]. To the best of our knowledge, our study represents the largest multi-center, population-based real-world dataset comparing ER-low and ER-zero breast cancer patients treated with neoadjuvant immuno-chemotherapy.

In our previous population-based study of Swedish patients diagnosed between 2008 and 2020, we demonstrated that ER-low breast cancer exhibited similar clinical characteristics and prognosis to ER-zero breast cancer when treated as TNBC [32]. These findings are concordant with our current results following the addition of pembrolizumab to the neoadjuvant regimen.

Our current population-based real-world findings are particularly timely in light of the recently published KEYNOTE-756 (KN756) [33] and CheckMate 7FL [34] trials, both of which demonstrated the benefit of adding pembrolizumab to neoadjuvant chemotherapy in high-risk, ER-positive /HER2-negative breast cancer. Notably, the trials also included ER-low tumors, 34 and 18 patients respectively, who exhibited substantially higher pCR rates than patients with ER-positive tumors, highlighting a gap in evidence for this biologically distinct subgroup. In our study, ER-low patients showed a comparable pCR rate of 58.1%, closely aligning with the 55.9% reported in KN756. Our results therefore help to address this gap by providing real-world data on the neoadjuvant immunotherapy responsiveness of ER-low tumors when treated as TNBC, suggesting that these tumors behave more like ER-negative disease when treated with immunotherapy.

In a recent population-based study involving over 10,000 ER-low patients, those who achieved a pCR after neoadjuvant chemotherapy demonstrated comparable prognoses regardless of whether they received adjuvant endocrine therapy or not [35]. This finding highlights that pCR is linked to improved prognosis in ER-low breast cancer, reinforcing the clinical relevance of increased pCR rates after adding neoadjuvant immunotherapy, demonstrated in our study.

The main strengths of our study include its nationwide, population-based design and the large cohort size comprising both ER-zero and ER-low HER2-negative patients treated as TNBC, with detailed real-world high-quality individual clinicopathological data and treatment response metrics, including RCB scores. Given Sweden’s tax-funded healthcare system, which includes a national breast cancer screening program for women aged 40–75, all patients within the specified time frame represent a diverse patient population, thereby enhancing the generalizability of our findings to similar populations. Nonetheless, several limitations should be acknowledged. First, despite the cohort size covering 99% of all Swedish breast cancers diagnosed during a 2-year period, ER-low disease remains rare, limiting statistical power. As a further step, the results of this study should ideally be validated in larger cohorts. Second, the short follow-up period precludes analysis of long-term prognostic outcomes. Third, the dataset lacks complete information on treatment regimens and completion rates, limiting the findings to an intention-to-treat population. Fourth, no central pathology review was performed, including ER status, Ki67 and sTILs. However, a vast majority of cases were primarily assessed by dedicated breast pathologists, with limited inter-laboratory variability [36]. 

## Conclusions

In conclusion, our findings indicate comparable pathological responses to neoadjuvant chemo-immunotherapy with pembrolizumab in both ER-zero and ER-low breast cancer patients. This supports previous evidence suggesting that ER-low tumors behave more like ER-zero disease. We believe this population-based study provides valuable real-world insights into the evolving classification and management of ER-low breast cancer and highlights the potential role of sTILs as a biomarker in the immunotherapy era, especially given the unlikelihood of sufficiently powered ongoing prospective trial specifically addressing ER-low breast cancer patients.

## Supplementary Information

Below is the link to the electronic supplementary material.

Supplementary Material 1

## Data Availability

The data are not publicly available due to restrictions by Swedish and European law, to protect patient privacy. Data are available from the register holder of the Swedish National Breast Cancer Quality Register for researchers with relevant ethical approvals and who meet the criteria for access to confidential data.

## Abbreviations

ASCO: American Society of Clinical Oncology
BC: Breast cancer
CAP: College of American Pathologists
χ^2^: Chi-squared
ER-low: Estrogen receptor-low
HER2: Human epidermal growth factor receptor 2
HER2-: HER2-negative
IHC: Immunohistochemistry
ISH: *in situ* Hybridization
KN522: KEYNOTE-522
KN756: KEYNOTE-756
LIS: Laboratory Information Systems
pCR: Pathological complete response
PR: Progesterone receptor
RCB: Residual cancer burden
sTILs: Stromal tumor-infiltrating lymphocytes
SweBCG: Swedish Breast Cancer Group
TILs: Tumor-infiltrating lymphocytes
TNBC: Triple-negative breast cancer
