# Supplementary material for: Pathological response to pembrolizumab-based neoadjuvant therapy in ER-low vs. ER-zero breast cancer: a Swedish population-based cohort study
Source: Breast Cancer Res. 2025 Nov 29;27:213. doi: 10.1186/s13058-025-02179-3 (PMC12670802; doi:10.1186/s13058-025-02179-3)
Supplement: Supplementary file 1 — Supplementary Material 1 [file 13058_2025_2179_MOESM1_ESM.pdf]

Supplementary materials

Pathological Response to Pembrolizumab-based Neoadjuvant Therapy in ER-Low vs ER-Zero Breast Cancer: A Swedish Population-Based Cohort Study

Table of contents

Table S1: Clinicopathological characteristics of the cohort based on treatment-naïve biopsies and surgical specimens……………………………………………………………………….. 2

Table S2: Crosstabulations of ER status with pCR and dichotomised RCB score, including chi-squared test results.…………………………………………………………………….. 3

Figure S1: Boxplots illustrating division of clinicopathological characteristics between oestrogen receptor (ER)-zero and ER-low groups…………………………………………… 4

Table S3: Crosstabulation of PR status by pCR with chi-squared test result………………… 5

Table S4: Multivariable logistic regression model with pathological complete response (pCR) as outcome variable ……………………………………………………………………..… 6

Figure S2: Cross-validation ROC curve……………………………………………………... 6

Table S1: Clinicopathological characteristics of the cohort based on treatment-naïve biopsies and surgical specimens

		Biopsy		Surgical specimen	
**Clinicopathological data**	**Parameters**	**N**	**(%)**	**N**	**(%)**
Cohort	Number of cases	441		441	
Histological subtype	Invasive breast carcinoma NST	395	(89.6)	155	(35.1)
	Invasive lobular carcinoma	5	(1.1)	5	(1.1)
	Invasive carcinoma NST and invasive lobular carcinoma	1	(0.2)	0	(0.0)
	Carcinoma with apocrine differentiation	6	(1.4)	4	(0.9)
	Metaplastic carcinoma	8	(1.8)	8	(1.8)
	Invasive carcinoma NOS	0	(0.0)	3	(0.7)
	Unclassifiable	8	(1.8)	1	(0.2)
	Not specified	18	(4.1)	24	(5.4)
	No residual invasive tumour	- -	- -	241	(54.6)
ER status	Negative/ER-zero (<0%)	398	(90.2)	153	(34.7)
	ER-low (1-9%)	43	(9.8)	11	(2.5)
	Positive (>9%)	0	(0.0)	12	(2.7)
	Not assessed	0	(0.0)	24	(5.4)
	No residual invasive tumour	- -	- -	241	(54.6)
PR status	Negative (<0%)	407	(92.3)	168	(38.1)
	PR-low (1-9%)	25	(5.7)	5	(1.1)
	Positive (>9%)	8	(1.8)	3	(0.7)
	Not assessed	1	(0.2)	24	(5.4)
	No residual invasive tumour	- -	- -	241	(54.6)
Ki67	High (>29%)	397	(90.0)	83	(18.8)
	Intermediate (6-29%)	37	(8.4)	44	(10.0)
	Low (<6%)	1	(0.2)	46	(10.4)
	Inconclusive	0	(0.0)	2	(0.5)
	Not assessed	6	(1.4)	25	(5.7)
	No residual tumour	- -	- -	241	(54.6)
HER2 status	Negative	440	(99.8)	154	(36.6)
	Positive	0	(0.0)	3	(0.7)
	Not specified	1	(0.2)	1	(0.2)
	Not assessed	0	(0.0)	0	(0.0)
	No residual invasive tumour	- -	- -	241	(57.2)
Nottingham Histological Grade	NHG 1	1	(0.2)	8	(1.8)
	NHG 2	64	(14.5)	78	(17.7)
	NHG 3	342	(77.6)	75	(17.0)
	Inconclusive	0	(0.0)	1	(0.2)
	Not assessed	34	(7.7)	37	(8.4)
	No residual invasive tumour	- -	- -	241	(54.8)
sTILs	<30%	205	(46.5)	- -	- -
	≥30%	132	(29.9)	- -	- -
	Not assessed	104	(23.6)	- -	- -
pCR	Yes	- -	- -	226	(51.2)
	No	- -	- -	212	(48.1)
	Inconclusive	- -	- -	3	(0.7)
RCB score	0	- -	- -	226	(51.2)
	1	- -	- -	42	(9.5)
	2	- -	- -	113	(25.6)
	3	- -	- -	37	(8.4)
	Not specified	- -	- -	23	(5.2)
Number of lymph node metastases	0	- -	- -	340	(81.0)
	1-3	- -	- -	70	(16.7)
	4-9	- -	- -	0	(0.0)
	≥10	- -	- -	10	(2.4)
	Data missing	- -	- -	21	(4.8)

ER = oestrogen receptor; PR = progesterone receptor; HER2 = human epidermal growth factor receptor 2; sTILs = stromal tumour-infiltrating lymphocytes; pCR = pathological complete response; RCB = residual cancer burden

Table S2: Crosstabulations of ER status with pCR and dichotomised RCB score, including chi-squared test results

ER status by pCR		
	ER-zero	ER-low
Yes	201	25
No	194	18
No. Obs. = 438; x^2 p = 0.46		

ER status by RCB low/high		
	ER-zero	ER-low
0-1 (low)	242	26
2-3 (high)	134	16
No. Obs. = 418; x^2 p = 0.88		

ER = oestrogen receptor; pCR = pathological complete response; RCB = residual cancer burden; No. = number; Obs. = observations

Figure S1: Boxplots illustrating division of clinicopathological characteristics between oestrogen receptor (ER)-zero and ER-low groups.

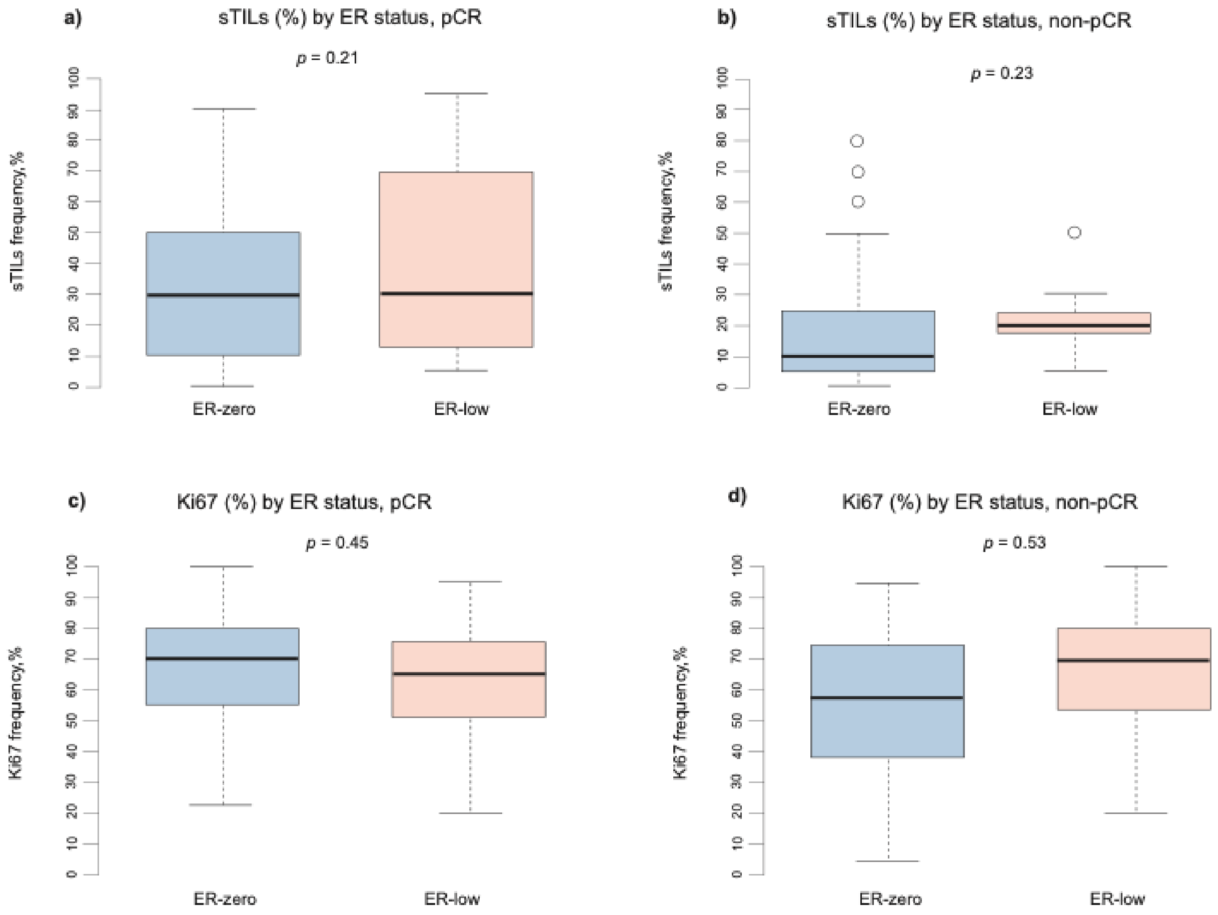

Subgroup analysis of stromal tumour-infiltrating lymphocytes (sTILs) scores by ER status (ER-zero vs ER-low) in patients with pathological complete response (pCR) (a) and without pCR (b), and the proliferation marker Ki67 index by ER status in pCR (c) and non-pCR (d) cases.

sTILs = stromal tumour-infiltrating lymphocytes; ER = oestrogen receptor; pCR = pathological complete response; Ki67 = proliferation marker

Table S3: Association between progesterone receptor (PR) status and pathological complete response (pCR) assessed by chi-squared test

PR status by pCR		
	<10%	≥10%
Yes	222	3
No	207	5
No. Obs. = 437; x^2 p = 0.66, Fisher's p = 0.49		

PR = progesterone receptor; pCR = pathological complete response; No. = number; Obs. = observations

Table S4: Multivariable logistic regression model with pathological complete response (pCR) as outcome variable

	Event N	OR	95% CI	*p*-value
Ki67	162	0.98	0.97, 0.99	0.0006
sTILs	162	0.98	0.97, 0.99	<0.0001
ER	162	0.87	0.70, 1.04	0.16
PR	162	1.02	0.93, 1.13	0.68

CI = Confidence Interval; OR = Odds Ratio; sTILs = stromal tumour-infiltrating lymphocytes; ER = oestrogen receptor; PR = progesterone receptor; Null deviance = 459; Null degrees of freedom (df)= 330; Log-likelihood = -207; AIC = 424; BIC = 443; Deviance = 414; Residual df = 326; No. Obs. = 331

In the multivariable logistic regression model, oestrogen receptor (ER), progesterone receptor (PR), proliferation marker (Ki67) and stromal tumour-infiltrating lymphocytes (sTILs) were included as continuous variables to evaluate their adjusted association with pathological complete response (pCR). High levels of sTILs and Ki67 were independently associated with pCR (p<0·0001 and p=0.0006, respectively), whereas ER and PR status showed no significant associations with pCR in the multivariable setting (p=0.16 and p=0.68, respectively).

Figure S2: Cross-validation Receiver Operating Characteristics (ROC)-curve

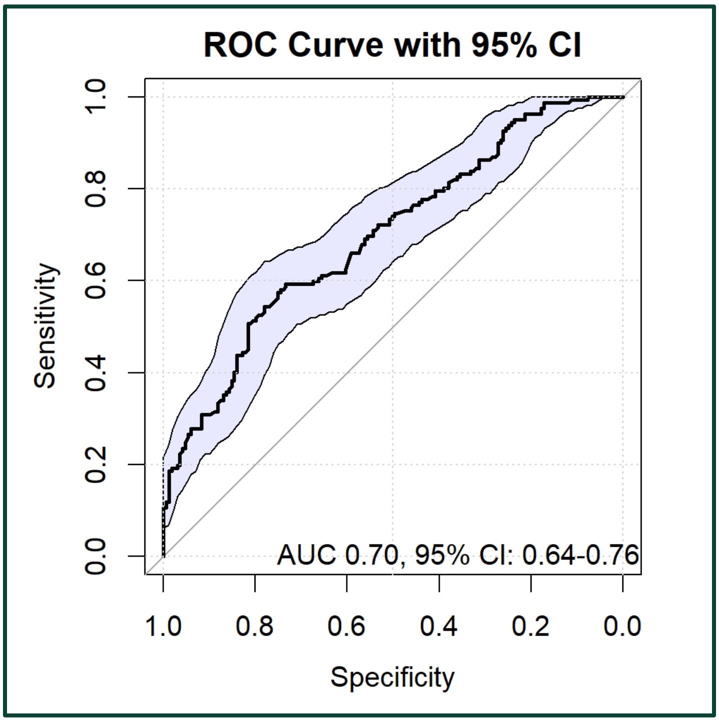

Cross-validation of the multivariable model yielded an area under the curve) (AUC) of 0·70 (95% confidence interval (CI): 0·64-0·76) for predicting pathological complete response (pCR).
